# Identification of the SIRT1 gene's most harmful non-synonymous SNPs and their effects on functional and structural features-an *in silico* analysis

**DOI:** 10.12688/f1000research.128706.1

**Published:** 2023-01-16

**Authors:** Desy Thayyil Menambath, Usha Adiga, Tirthal Rai, Sachidananda Adiga, Vijith Shetty

**Affiliations:** 1 Biochemistry, KS Hegde Medical Academy, NITTE (DU), Mangalore, Karnataka, 575018, India; 2 Pharmacology, KS Hegde Medical Academy, NITTE(DU), Mangalore, Karnataka, 575018, India; 3 Oncology, KS Hegde Medical Academy, NITTE(DU), Mangalore, Karnataka, 575018, India

**Keywords:** SIRT1, nsSNP, bioinformatics, protein modelling

## Abstract

**Introduction:** The sirtuin (Silent mating type information regulation 2 homolog)1(SIRT1) protein plays a vital role in many disorders such as diabetes, cancer, obesity, inflammation, and neurodegenerative and cardiovascular diseases. The objective of this *in silico* analysis of SIRT1's functional single nucleotide polymorphisms (SNPs) was to gain valuable insight into the harmful effects of non-synonymous SNPs (nsSNPs) on the protein. The objective of the study was to use bioinformatics methods to investigate the genetic variations and modifications that may have an impact on the SIRT1 gene's expression and function.

**Methods:** nsSNPs of SIRT1 protein were collected from the dbSNP site, from its three (3) different protein accession IDs. These were then fed to various bioinformatic tools such as SIFT, Provean, and I- Mutant to find the most deleterious ones. Functional and structural effects were examined using the HOPE server and I-Tasser. Gene interactions were predicted by STRING software. The SIFT, Provean, and I-Mutant tools detected the most deleterious three nsSNPs (rs769519031, rs778184510, and rs199983221).

**Results:** Out of 252 nsSNPs, SIFT analysis showed that 94 were deleterious, Provean listed 76 dangerous, and I-Mutant found 66 nsSNPs resulting in lowered stability of proteins. HOPE modelling of rs199983221 and rs769519031 suggested reduced hydrophobicity due to Ile 4Thr and Ile223Ser resulting in decreased hydrophobic interactions. In contrast, on modelling rs778184510, the mutant protein had a higher hydrophobicity than the wild type.

**Conclusions:** Our study reports that three nsSNPs (D357A, I223S, I4T) are the most damaging mutations of the SIRT1 gene. Mutations may result in altered protein structure and functions. Such altered protein may be the basis for various disorders. Our findings may be a crucial guide in establishing the pathogenesis of various disorders.

## Introduction

### *In silico* analysis of SIRT1 Gene

Sirtuins are nicotinamide adenine dinucleotide (NAD+)-dependent deacetylases that regulate transcriptional activity intracellularly. They are present in a wide range of tissues, such as the adipose, kidney, brain, liver, and muscle tissues. ^1^^,^^2^ SIRT1 (Silent mating-type information regulation 2 homolog 1) is known to regulate a variety of cellular processes, including lipid and glucose metabolism, stress tolerance, autophagy, circadian rhythms, and mitochondrial biogenesis, according to several studies. ^3^^–^^5^ SIRT1 gene expression modulates its downstream pathways in diabetes, cancer, obesity, inflammation, and neurodegenerative and cardiovascular diseases by focusing on numerous cellular proteins, including nuclear factor-κB (NF-κB), endothelial nitric oxide synthase (eNOS), forkhead transcriptional factors (FoxOs), AMP-activated protein kinase (AMPK), protein tyrosine phosphatase (PTP). However, considerable evidence suggests that in a variety of malignant cell types, SIRT1 is upregulated and that SIRT1 antagonists prevented the development of cancer cells. ^6^^–^^8^

Single nucleotide polymorphisms (SNPs) are variations in the DNA sequence that result from the alterations in a single nucleotide (A, T, C, or G). Around 90% of human genetic variation is made up of SNPs. The three-billion-base human genome has SNPs at every 100–300 bases, with varying densities between regions. ^9^ The genome's coding and noncoding sections can both present SNPs. SNPs can have a wide spectrum of effects on how cells behave, from having no effect to causing disease or altering the reaction to a drug. Since they are responsible for about half of the genetic differences associated with human hereditary diseases, non-synonymous SNPs (nsSNPs) that result in an amino acid residue substitution in the protein product are also of high relevance. ^10^ There may be effects on transcription factor binding, splicing, or gene expression from coding synonymous SNPs (sSNPs) and SNPs that aren't in the gene promoter or coding regions. ^11^^,^^12^

Human reactions to viruses, medications, vaccinations, and other agents are significantly influenced by SNPs. SNPs are therefore useful in biomedical research, the creation of pharmaceutical products, the improvement of medical diagnostics, and the application of personalised medicine. ^13^ SNPs are responsible for specific phenotypes and therefore it is very important to identify them. This is a difficult task as it necessitates repeatedly evaluating thousands of SNPs in candidate genes. Selecting a group of SNPs for a study to determine the role of an SNP in a disease is a challenging endeavour; in these situations, a bioinformatics tool may be very helpful to distinguish between neutral and functional SNPs. They might also show the structural underpinnings of the mutations. These bioinformatics applications are used to assess the SNPs’ functional significance.

To find the SIRT1 protein's most dangerous nsSNPs, we applied bioinformatics techniques. We hypothesised that SIRT1 protein would be harmful because of nsSNPs on the gene. This is the first study of its kind for the SIRT1 gene to include both protein structure prediction and mutation analysis.

## Methods

**Figure 1.**
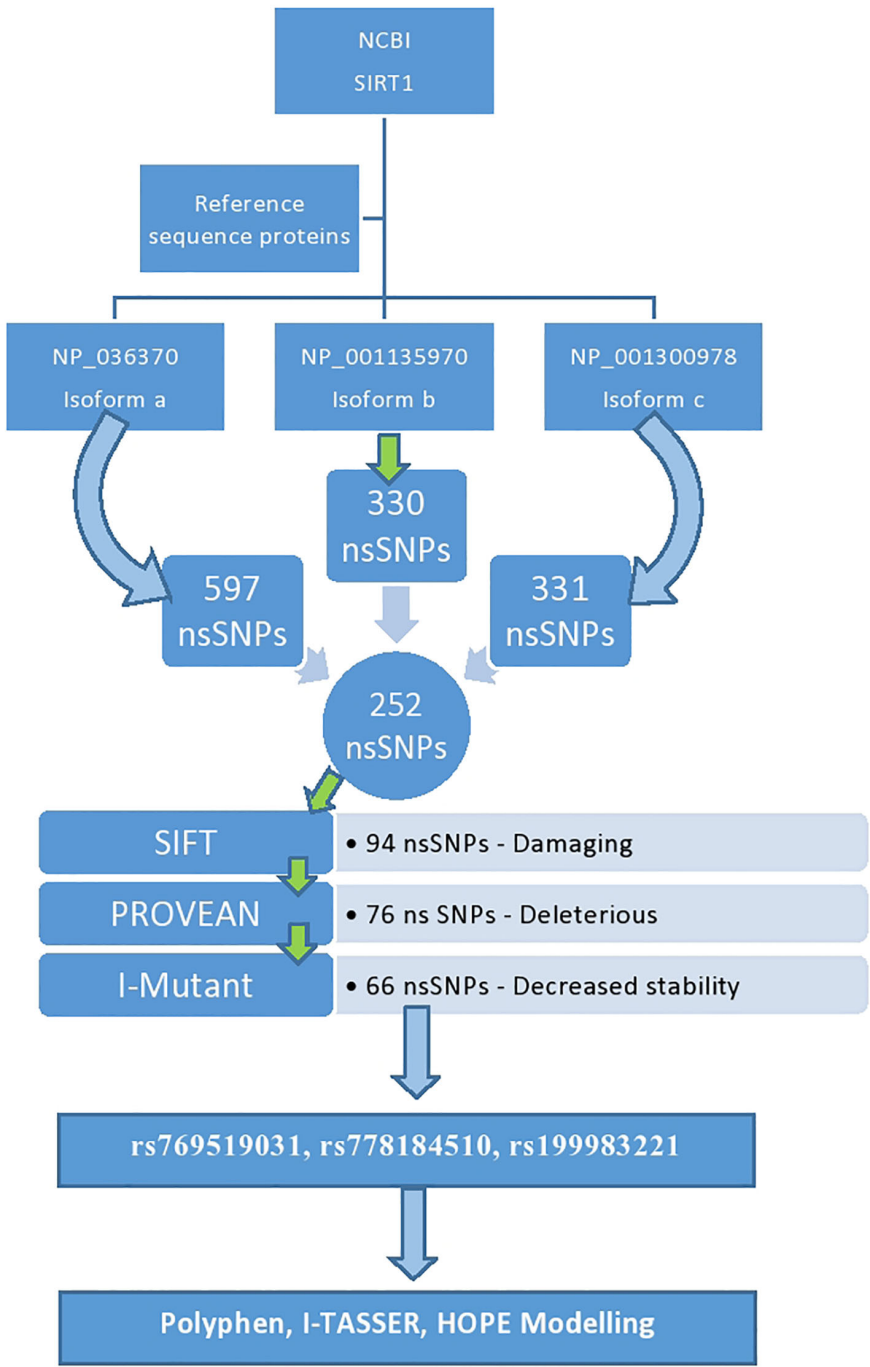
Sorting of nsSNPs of SIRT1.

Multiple steps were used to complete the study. The figure below shows the equipment used to complete the task ( Figure 1).

### Extraction of nsSNPs

The NCBI SNP database was accessed. Information on the entire SIRT1 gene, including its nsSNPs, was obtained. As the query sequence, filtered nsSNPs from the dbSNP database were examined. The NCBI Protein accession IDs NP_ 036370.2, NP_001135970.1, and NP_002294.2 for the SIRT1 gene were used.

### Identification of damaging nsSNPs

SIFT, Provean, and I-Mutant software were used to identify the impact of spotted nsSNPs on the SIRT1 gene.

*SIFT (Sorting Intolerant from Tolerant) server*

The SIFT server is a web-based bioinformatics tool that forecasts the detrimental effects of nucleotide substitution and frame shift (insertion/deletion) on protein function based on the degree of amino acid residue maintenance in sequence alignments obtained from highly associated sequences, with the primary assumption that mutations in evolutionarily conserved regions primarily affect its function. ^14^ The distinct input data order for the SIFT server includes protein sequence, chromosome location, and dbSNP reference number. SNPs and Indels were separated from the overall number in order to use this tool, and they were provided with the chromosome positions for frame shift indels and the residue number (rs) ID numbers for missense, nonsense, and stop gain SNPs. Each residue was given a value from 0 to 1 by the SIFT server, with scores below 0.05 indicating detrimental amino acid changes and scores above 0.05 indicating tolerance. ^15^ The website hosts SIFT version 5.2.2.

*Provean*

A protein's biological activity may be impacted by an amino acid substitution or indel, according to predictions made by the programme Protein Variation Effect Analyzer (PROVEAN). When filtering sequence variants, PROVEAN is useful for locating nonsynonymous or indel variations that are anticipated to be functionally significant. ^16^ The tool takes as input a protein sequence and several amino acid combinations, runs a BLAST search to find related sequences (supporting sequences), and outputs PROVEAN scores. The interpretation was done using the score thresholds. The default threshold is -2.5, meaning that variants with a score of -2.5 or less are deemed harmful, whereas variants with a score of -2.5 or more are considered neutral. http://provean.jcvi.org/index.php can be visited to access Provean.

*I-Mutant 2.0*

I-Mutant 2.0 was used in the investigation to analyse the stability of the targeted SIRT1 protein. This website server estimates any mutation-related changes to protein stability. ^17^ By adjusting the pH to 7 and the temperature to 25°C, this technique was used to analyse the SIRT1 protein sequences. It gives the opportunity to forecast how the protein's stability will be altered in response to single-site changes in the protein's structure or sequence. The design of the I-Mutant outcome is as follows: Free energy change value; Delta Delta G (DDG)= 0 is neutral, DDG > 0 is an increase in stability, and DDG <0 is a reduction in stability (I-Mutant website)

### Examining the functional and structural effect of nsSNPs

To understand the effect of nsSNPs on the SIRT1 protein structure, the study used Polyphen, HOPE and I-Tasser software. The three most deleterious and damaging nsSNPs of the SIRT1 gene from each of its isoforms were chosen and processed to examine their structural and functional effects on SIRT1 protein.

*Polyphen*

Using simple physical and comparative considerations, polymorphism phenotyping v2(PolyPhen) is a method that estimates the potential effects of an amino acid substitution on the structure and functionality of a human protein. ^18^

*HOPE modelling*

The HOPE server analyses mutations automatically and can show the structural repercussions of a mutation. In addition to predictions from DAS services, sequence annotations from the UniProt database and calculations on the 3D coordinates of the protein using WHAT IF Web services are just a few of the data sources that HOPE uses to compile its information. ^19^

Iterative Threading ASSEmbly Refinement ( *I-Tasser)*

I-Tasser is a software package for protein structure and function modelling. The Template modelling score was used to compare the wild and mutant models. The estimated values of root mean square deviation (RMSD) and melting temperature (TM) allowed for the precise determination of similarity score. According to statistics, a TM-score of 0.17 or less indicates that two randomly chosen structures from the Protein DataBank library are comparable, while a score of 0.5 or more indicates that two structures have a similar topology. Studies have demonstrated a clear correlation between a high level of RMSD value and a high amount of change between wild-type and mutant. ^20^^,^^21^ The harmful mutations were then introduced into I-Tasser by adhering to their values. ^22^^–^^24^ Chimera 1.11 was used to study the molecular characteristics and interactive visualisation of the final protein structure. ^25^

### Gene-gene interactions of the SIRT1 gene

Managing protein interactions is essential for maintaining the system's homeostasis. STRING's task is to display the total score of interaction genes. In this stage, SIRT1 served as the input target gene, and analysis was completed.

## Results and discussion

### Extraction of nsSNPs

The number of SNPs of the SIRT1 gene obtained from the NCBI database were 15,865. Three isoforms for SIRT1 were found ( isoform a, isoform b and isoform c). Isofrom a (NP_036370.2) comprised 597 nsSNPs, isoform b (NP_001135970.1) comprised a total of 330 nsSNPs and isoform c (NP_001300978.1) 331 nsSNPs. The diagrammatical depiction is shown in Figure 1. The nsSNPs listed in all the isoforms are listed and the duplicates were deleted; the total number of nsSNPs included in all the isoforms was 252 ( Table 1).

**Table 1.** SIFT, I-Mutant, and Provean analyses for the nsSNPs of the SIRT1 Gene.

Coordinates	Known protein ID	Substitution	dbSNP ID	Prediction	SIFT score	DDG (Kcal/mol/stability)	Provean score	prediction
10,69647216,1,C/T	Q96EB6	H158Y	rs767540399	TOLERATED	1	0.06/increased	-1.115	Neutral
10,69648720,1,A/G	Q96EB6	I210V	rs369379188	TOLERATED	0.82	-0.61/decreased	0.333	Neutral
10,69644769,1,C/A	Q96EB6	A97E	rs550317521	TOLERATED	0.76	-0.21/decreased	-0.457	Neutral
10,69647188,1,C/A	Q96EB6	F148L	rs773494159	TOLERATED	0.75	-1.27/decreased	0.215	Neutral
10,69644762,1,A/T	Q96EB6	T95S	rs767360333	TOLERATED	0.75	-0.03/decreased	-0.254	Neutral
10,69647286,1,G/A	Q96EB6	R181Q	rs547102478	TOLERATED	0.72	-1.13/decreased	0.115	Neutral
10,69648649,1,C/G	Q96EB6	T186S	rs201841214	TOLERATED	0.64	-0.59/decreased	0.061	Neutral
10,69644828,1,G/A	Q96EB6	A117T	rs756786838	TOLERATED	0.59	-0.77/decreased	0.599	Neutal
10,69644675,1,G/C	Q96EB6	G66R	rs531048058	TOLERATED	0.59	-0.20/decreased	0.25	Neutral
10,69644864,1,G/A	Q96EB6	G129S	rs746381892	TOLERATED	0.53	-0.58/decreased	-0.191	Neutral
10,69648723,1,C/A	Q96EB6	P211T	rs201007799	TOLERATED	0.51	-0.92/decreased	-0.014	Neutral
10,69647222,1,T/A	Q96EB6	C160S	rs763883280	TOLERATED	0.49	-0.82/decreased	0.042	Neutral
10,69644778,1,A/G	Q96EB6	E100G	rs199624804	TOLERATED	0.49	-0.73/decreased	-1.003	Neutral
10,69648724,1,C/T	Q96EB6	P211L	rs201668639	TOLERATED	0.48	-0.43/decreased	1.077	Neutral
10,69647273,1,A/G	Q96EB6	T177A	rs565217830	TOLERATED	0.47	-1.23/decreased	-0.59	Neutral
10,69644820,1,C/A	Q96EB6	P114Q	rs182199697	TOLERATED	0.45	-1.37/decreased	-0.658	Neutral
10,69648744,1,A/G	Q96EB6	M218V	rs745979871	TOLERATED	0.4	-0.63/decreased	-1.14	Neutral
10,69644688,1,C/T	Q96EB6	A70V	rs751564985	TOLERATED	0.37	-0.05/decreased	0.69	Neutral
10,69647183,1,C/G	Q96EB6	L147V	rs748384143	TOLERATED	0.34	-1.7/decreased	-0.174	Neutral
10,69648722,1,A/G	Q96EB6	I210M	rs201862792	TOLERATED	0.32	-1.39/decreased	-0.025	Neutral
10,69647233,1,T/A	Q96EB6	D163E	rs761508157	TOLERATED	0.31	-0.06/decreased	-1.813	Neutral
10,69644519,1,T/C	Q96EB6	S14P	rs201230502	TOLERATED	0.27	0.06/increased	-0.631	Neutral
10,69647181,1,T/C	Q96EB6	L146P	rs772106776	TOLERATED	0.26	-1.89/decreased	-0.95	Neutral
10,69644589,1,C/T	Q96EB6	P37L	rs548590752	TOLERATED	0.26	-0.27/decreased	0.112	Neutral
10,69648765,1,A/G	Q96EB6	I225V	rs559057403	TOLERATED	0.22	-1.10/decreased	-0.362	Neutral
10,69648669,1,A/G	Q96EB6	M193V	rs762526864	TOLERATED	0.22	-0.49/decreased	-0.716	Neutral
10,69647253,1,A/G	Q96EB6	H170R	rs144124002	TOLERATED	0.21	-0.19/decreased	-1.457	Neutral
10,69644799,1,T/C	Q96EB6	L107P	rs587776957	TOLERATED	0.21	-1.31/decreased	-1.226	Neutral
10,69647175,1,A/G	Q96EB6	D144G	rs142378619	TOLERATED	0.2	-0.61/decreased	1.231	Neutral
10,69647219,1,T/C	Q96EB6	S159P	rs200660028	TOLERATED	0.2	-0.40/decreased	-1.69	Neutral
10,69647276,1,C/G	Q96EB6	P178A	rs752035789	TOLERATED	0.17	E	-2.017	Neutral
10,69648861,1,A/G	Q96EB6	I257V	rs765326045	TOLERATED	0.14	-0.79/decreased	-0.642	Neutral
10,69648670,1,T/C	Q96EB6	M193T	rs200994303	TOLERATED	0.12	-0.81/decreased	-1.398	Neutral
10,69648666,1,C/G	Q96EB6	L192V	rs772852024	TOLERATED	0.11	-1.28/decreased	-0.843	Neutral
10,69647289,1,T/G	Q96EB6	I182R	rs749341245	TOLERATED	0.09	-1.63/decreased	-0.979	Neutral
10,69644622,1,C/T	Q96EB6	P48L	rs568432780	TOLERATED	0.08	-0.20/decreased	-0.384	Neutral
10,69648819,1,A/G	Q96EB6	I243V	rs200711525	TOLERATED	0.06	-1.08/decreased	-0.89	Neutral
10,69644886,1,C/G	Q96EB6	A136G	rs775978922	DAMAGING	0.05	-1.07/decreased	-0.329	Neutral
10,69648718,1,C/G	Q96EB6	T209R	rs756483371	DAMAGING	0.05	-0.16/decreased	-2.574	Deleterious
10,69648745,1,T/C	Q96EB6	M218T	rs201583175	DAMAGING	0.04	-0.70/decreased	-1.991	Neutral
10,69647279,1,A/G	Q96EB6	R179G	rs200805107	DAMAGING	0.04	-1.66/decreased	-1.894	Neutral
10,69647220,1,C/A	Q96EB6	S159Y	rs760482544	DAMAGING	0.04	-0.23/decreased	-2.272	Neutral
10,69647264,1,A/C	Q96EB6	S174R	rs758805613	DAMAGING	0.04	-0.27/decreased	-2.166	Neutral
10,69651254,1,C/G	Q96EB6	A295G	rs368002483	DAMAGING	0.01	-1.33/decreased	-3.508	Deleterious
10,69647193,1,A/T	Q96EB6	D150V	rs776925394	DAMAGING	0.01	-0.13/decreased	-2.979	Deleterious
10,69651200,1,A/G	Q96EB6	D277G	rs776933716	DAMAGING	0.01	-1.50/decreased	-4.86	Deleterious
10,69651236,1,A/G	Q96EB6	D289G	rs375090685	DAMAGING	0.01	-0.90/decreased	-5.977	Deleterious
10,69644516,1,G/T	Q96EB6	G13C	rs200005116	DAMAGING *Warning! Low confidence.	0.01	-0.64/decreased	-0.496	Neutral
10,69648820,1,T/C	Q96EB6	I243T	rs776084517	DAMAGING	0.01	-1.91/decreased	-2.831	Deleterious
10,69648712,1,C/T	Q96EB6	P207L	rs375988661	DAMAGING	0.01	-0.48/decreased	-6.177	Deleterious
10,69648786,1,C/A	Q96EB6	P232T	rs750787952	DAMAGING	0.01	-0.98/decreased	-4.805	Deleterious
10,69648748,1,C/G	Q96EB6	T219R	rs199716245	DAMAGING	0.01	-0.24/decreased	-4.312	Deleterious
10,69651253,1,G/A	Q96EB6	A295T	rs751811485	DAMAGING	0	-0.81/decreased	-3.491	Deleterious
10,69648827,1,T/A	Q96EB6	D245E	rs201479376	DAMAGING	0	-0.28/decreased	-3.358	Deleterious
10,69648825,1,G/C	Q96EB6	D245H	rs761327480	DAMAGING	0	-0.41/decreased	-5.393	Deleterious
10,69644488,1,C/A	Q96EB6	D3E	rs35671182	DAMAGING *Warning! Low confidence.	0	0.59/increase	-0.464	Neutral
10,69648760,1,T/G	Q96EB6	I223S	rs769519031	DAMAGING	0	-2.25/decreased	-4.47	Deleterious
10,69648811,1,T/C	Q96EB6	I240T	rs199618656	DAMAGING	0	-1.77/decreased	-3.43	Deleterious
10,69648867,1,C/G	Q96EB6	L259V	rs750671807	DAMAGING	0	-1.35/decreased	-2.769	Deleterious
10,69651218,1,T/G	Q96EB6	L283R	rs773632625	DAMAGING	0	-2/decreased	-5.537	Deleterious
10,69651215,1,G/A	Q96EB6	R282H	rs762393274	DAMAGING	0	-1.60/decreased	-4.481	Deleterious
10,69651193,1,T/A	Q96EB6	S275T	rs779685735	DAMAGING	0	-0.67/decreased	-2.769	Deleterious
10,69651285,1,T/G	NP_001135970	D10E	rs780983084	DAMAGING	0.01	-0.52/decreased	-3.322	Deleterious
10,69651266,1,T/C	NP_001135970	I4T	rs199983221	DAMAGING	0	-2.2/decreased	-4.39	Deleterious
10,69651258,1,G/T	NP_001135970	M1I	rs753708973	DAMAGING	0	-0.17/decreased	-3.705	Deleterious
10,69651292,1,C/G	NP_001135970	P13A	rs200902165	DAMAGING	0	-1.26/decreased	-7.539	Deleterious
10,69672378,1,G/A	B0QZ35	C199Y	rs150099719	TOLERATED	1	-0.64/decreased	-1.163	Neutral
10,69676275,1,T/A	B0QZ35	D420E	rs775109357	TOLERATED	1	-0.31/decreased	-0.41	Neutral
10,69676297,1,A/G	B0QZ35	I428V	rs35224060	TOLERATED	1	0.33/increased	0.108	Neutral
10,69669103,1,C/G	B0QZ35	Q118E	rs746762337	TOLERATED	1	-1.24/decreased	0.07	Neutral
10,69666602,1,C/T	B0QZ35	S30L	s200610338	TOLERATED	1	E	3.993	Neutral
10,69667859,1,G/A	B0QZ35	V80I	rs750895479	TOLERATED	1	0.37/increased	0.385	Neutral
10,69672512,1,C/T	B0QZ35	P244S	rs201723648	TOLERATED	0.94	E	0.648	Neutral
10,69672620,1,C/A	B0QZ35	Q280K	rs769257926	TOLERATED	0.92	-0.94/decreased	-0.787	Neutral
10,69672599,1,G/A	B0QZ35	E273K	rs779678154	TOLERATED	0.85	-1.3/decreased	-1.264	Neutral
10,69669016,1,G/A	B0QZ35	V89I	rs201152568	TOLERATED	0.85	-0.27/decreased	0.201	Neutral
10,69672597,1,T/C	B0QZ35	M272T	rs757828571	TOLERATED	0.81	-0.33//decreased	-0.187	Neutral
10,69676028,1,A/G	B0QZ35	Q338R	rs766102594	TOLERATED	0.81	-1.7/decreased	-1.453	Neutral
10,69672692,1,A/G	B0QZ35	T304A	rs779413432	TOLERATED	0.8	-0.83/decreased	-0.746	Neutral
10,69672720,1,T/C	B0QZ35	V313A	rs781614748	TOLERATED	0.76	-1.24/decreased	-0.056	Neutral
10,69672590,1,G/A	B0QZ35	G270S	rs144625497	TOLERATED	0.73	-0.58/decreased	0.377	Neutral
10,69676289,1,A/G	B0QZ35	N425S	rs761406151	TOLERATED	0.72	-0.12/decreased	-0.158	Neutral
10,69672755,1,G/C	B0QZ35	V325L	rs567829185	TOLERATED	0.71	-0.86/decreased	-0.246	Neutral
10,69672683,1,G/A	B0QZ35	G301S	rs200296961	TOLERATED	0.67	-0.77/decreased	0.011	Neutral
10,69672680,1,G/C	B0QZ35	V300L	rs750092788	TOLERATED	0.62	-0.18/decreased	-0.652	Neutral
10,69672383,1,C/A	B0QZ35	P201T	rs116499760	TOLERATED	0.59	-0.28/decreased	-0.581	Neutral
10,69672605,1,A/C	B0QZ35	K275Q	rs747447296	TOLERATED	0.58	-0.69/decreased	-1.15	Neutral
10,69672684,1,G/A	B0QZ35	G301D	rs533321736	TOLERATED	0.57	-0.55/decreased	0.908	Neutral
10,69672243,1,A/G	B0QZ35	H154R	rs146837595	TOLERATED	0.57	-0.17/decreased	-1.947	Neutral
10,69672479,1,G/A	B0QZ35	E233K	rs116040871	TOLERATED	0.54	-1.18/decreased	-0.433	Neutral
10,69672675,1,A/G	B0QZ35	K298R	rs765178020	TOLERATED	0.52	-0.11/decreased	-0.612	Neutral
10,69672597,1,T/A	B0QZ35	M272K	rs757828571	TOLERATED	0.52	-0.31/decreased	-1.385	Neutral
10,69672539,1,T/G	B0QZ35	L253V	rs377735046	TOLERATED	0.51	-0.27/decreased	0.246	Neutral
10,69672495,1,C/G	B0QZ35	P238R	rs768902657	TOLERATED	0.51	0	-2.575	Deleterious
10,69672426,1,A/G	B0QZ35	Q215R	rs201058854	TOLERATED	0.49	-1.37/decreased	-1.134	Neutral
10,69672630,1,G/C	B0QZ35	R283T	rs773365044	TOLERATED	0.49	-0.44/decreased	0.351	Neutral
10,69676289,1,A/C	B0QZ35	N425T	rs761406151	TOLERATED	0.48	-0.03/decreased	-0.034	Neutral
10,69672438,1,C/T	B0QZ35	A219V	rs61754500	TOLERATED	0.44	-0.82/decreased	-0.93	Neutral
10,69672644,1,A/G	B0QZ35	I288V	rs370128548	TOLERATED	0.42	-0.19/decreased	0.248	Neutral
10,69669104,1,A/G	B0QZ35	Q118R	rs371326132	TOLERATED	0.42	-1.07/decreased	-1.537	Neutral
10,69672357,1,G/A	B0QZ35	G192D	rs369274325	TOLERATED	0.41	-0.2/decreased	-2.13	Neutral
10,69672380,1,A/G	B0QZ35	N200D	rs201090108	TOLERATED	0.41	-0.01/decreased	-1.016	Neutral
10,69672657,1,T/C	B0QZ35	M292T	rs139635382	TOLERATED	0.39	0.26/increased	-0.466	Neutral
10,69676240,1,G/A	B0QZ35	A409T	rs114182972	TOLERATED	0.38	-0.23/decreased	-0.877	Neutral
10,69672593,1,T/G	B0QZ35	C271G	rs745527350	TOLERATED	0.38	-2.81/decreased	-1.457	Neutral
10,69672335,1,G/C	B0QZ35	E185Q	rs771126114	TOLERATED	0.38	-0.9/decreased	-0.676	Neutral
10,69676099,1,G/A	B0QZ35	V362I	rs201730062	TOLERATED	0.38	-0.66/decreased	-0.29	Neutral
10,69676168,1,C/T	B0QZ35	P385S	rs757740493	TOLERATED	0.37	-0.82/decreased	-0.649	Neutral
10,69672564,1,A/G	B0QZ35	D261G	s756116247	TOLERATED	0.36	E	-0.904	Neutral
10,69672567,1,A/G	B0QZ35	D262G	rs763773932	TOLERATED	0.36	E	-1.552	Neutral
10,69669136,1,G/A	B0QZ35	E129K	rs763496433	TOLERATED	0.35	-0.86/decreased	-1.352	Neutral
10,69672456,1,C/T	B0QZ35	P225L	rs770755510	TOLERATED	0.35	0.36/increased	-1.813	Neutral
10,69667871,1,A/G	B0QZ35	I84V	rs17855431	TOLERATED	0.34	-0.28/decreased	-0.432	Neutral
10,69672377,1,T/C	B0QZ35	C199R	rs202116390	TOLERATED	0.33	-0.43/decreased	-3.055	Deleterious
10,69672632,1,A/G	B0QZ35	N284D	rs763220339	TOLERATED	0.33	-1.22/decreased	-0.461	Neutral
10,69667878,1,A/G	B0QZ35	N86S	rs17855432	TOLERATED	0.33	-0.7/decreased	-0.927	Neutral
10,69667859,1,G/C	B0QZ35	V80L	rs750895479	TOLERATED	0.33	0.19/increased	-0.68	Neutral
10,69672552,1,C/G	B0QZ35	A257G	rs762955289	TOLERATED	0.32	-3/decreased	-0.793	Neutral
10,69676118,1,G/C	B0QZ35	C368S	rs114575266	TOLERATED	0.32	-0.76/decreased	-2.036	Neutral
10,69676243,1,G/C	B0QZ35	G410R	rs771866041	TOLERATED	0.32	-0.14/decreased	-0.936	Neutral
10,69672621,1,A/C	B0QZ35	Q280P	rs201096600	TOLERATED	0.32	-0.63/decreased	-1.255	Neutral
10,69672456,1,C/A	B0QZ35	P225Q	rs770755510	TOLERATED	0.31	E	-1.222	Neutral
10,69676231,1,C/A	B0QZ35	P406T	rs770824011	TOLERATED	0.31	-0.66/decreased	-1.383	Neutral
10,69672684,1,G/T	B0QZ35	G301V	rs533321736	TOLERATED	0.3	-0.13/decreased	-0.94	Neutral
10,69676190,1,T/C	B0QZ35	I392T	rs140030776	TOLERATED	0.3	-0.26/decreased	-0.248	Neutral
10,69672490,1,T/G	B0QZ35	S236R	rs192990424	TOLERATED	0.3	-0.99/decreased	-1.914	Neutral
10,69672629,1,A/G	B0QZ35	R283G	rs202044007	TOLERATED	0.29	-1.1/decreased	-1.032	Neutral
10,69669023,1,G/A	B0QZ35	R91Q	rs766737138	TOLERATED	0.29	-0.85/decreased	-1.062	Neutral
10,69676297,1,A/C	B0QZ35	I428L	rs35224060	TOLERATED	0.28	0.72/increased	-0.316	Neutral
10,69672622,1,A/C	B0QZ35	Q280H	rs777147954	TOLERATED	0.28	-1.18/decreased	-1.047	Neutral
10,69669058,1,A/G	B0QZ35	I103V	rs757140711	TOLERATED	0.27	-0.32/decreased	-0.427	Neutral
10,69667830,1,T/G	B0QZ35	I70S	rs763566753	TOLERATED	0.27	E	0.432	Neutral
10,69672662,1,A/G	B0QZ35	N294D	rs753828684	TOLERATED	0.27	-0.4/decreased	-0.577	Neutral
10,69676282,1,G/T	B0QZ35	A423S	rs776051187	TOLERATED	0.26	-0.93/decreased	-0.122	Neutral
10,69672488,1,A/G	B0QZ35	S236G	rs199497583	TOLERATED	0.26	-2.12/decreased	-1.407	Neutral
10,69672369,1,A/G	B0QZ35	K196R	rs549636735	TOLERATED	0.25	-0.15/decreased	-0.838	Neutral
10,69672430,1,A/T	B0QZ35	K216N	rs200415719	TOLERATED	0.25	-0.97/decreased	-0.117	Neutral
10,69676231,1,C/T	B0QZ35	P406S	rs770824011	TOLERATED	0.25	-0.84/decreased	-1.273	Neutral
10,69672504,1,C/G	B0QZ35	T241S	rs777098039	TOLERATED	0.25	-0.74/decreased	-0.77	Neutral
10,69676286,1,T/C	B0QZ35	I424T	rs202021325	TOLERATED	0.23	E	-0.394	Neutral
10,69676298,1,T/C	B0QZ35	I428T	rs758781892	TOLERATED	0.23	0.03/increased	-0.426	Neutral
10,69676169,1,C/A	B0QZ35	P385H	rs765609303	TOLERATED	0.23	-0.92/decreased	-1.139	Neutral
10,69666569,1,A/G	B0QZ35	Q19R	rs532201569	TOLERATED	0.22	-0.1/decreased	-1.062	Neutral
10,69676276,1,C/G	B0QZ35	Q421E	rs201854199	TOLERATED	0.22	-0.33/decreased	-0.47	Neutral
10,69676238,1,G/C	B0QZ35	R408T	rs774376548	TOLERATED	0.22	-0.42/decreased	-0.622	Neutral
10,69676156,1,A/G	B0QZ35	S381G	rs114572830	TOLERATED	0.22	-1.11/decreased	-1.35	Neutral
10,69672755,1,G/A	B0QZ35	V325M	rs567829185	TOLERATED	0.21	-1.01/decreased	-0.598	Neutral
10,69669028,1,C/T	B0QZ35	P93S	rs752020872	TOLERATED	0.2	-1.86/decreased	-5.682	Deleterious
10,69666581,1,G/C	B0QZ35	C23S	rs200987359	TOLERATED	0.19	-1.24/decreased	-6.059	Deleterious
10,69672453,1,T/G	B0QZ35	L224W	rs748917510	TOLERATED	0.19	0.09/increased	-1.771	Neutral
10,69676205,1,A/G	B0QZ35	N397S	rs116459300	TOLERATED	0.19	-0.44//decreased	-1.479	Neutral
10,69672369,1,A/C	B0QZ35	K196T	rs549636735	TOLERATED	0.18	-0.26/decreased	-2.109	Neutral
10,69676340,1,A/G	B0QZ35	N442S	rs756220511	TOLERATED	0.18	-1.64/decreased	-0.75	Neutral
10,69676075,1,G/T	B0QZ35	V354L	rs199502996	TOLERATED	0.18	-0.53/decreased	-1.596	Neutral
10,69667850,1,T/G	B0QZ35	C77G	rs779440453	TOLERATED	0.17	-2.97/decreased	-3.709	Deleterious
10,69669041,1,C/G	B0QZ35	A97G	rs755416990	TOLERATED	0.16	-2.16/decreased	-0.743	Neutral
10,69669041,1,C/T	B0QZ35	A97V	rs755416990	TOLERATED	0.16	E	-0.179	Neutral
10,69676087,1,T/C	B0QZ35	S358P	rs754313614	TOLERATED	0.15	0.18/increased	-0.896	Neutral
10,69669139,1,G/A	B0QZ35	V130I	rs745364339	TOLERATED	0.15	-0.14/decreased	-0.482	Neutral
10,69676075,1,G/A	B0QZ35	V354I	rs199502996	TOLERATED	0.15	-0.27/decreased	-0.369	Neutral
10,69676303,1,G/A	B0QZ35	V430M	rs766908589	TOLERATED	0.15	0.45/increased	-0.04	Neutral
10,69676169,1,C/G	B0QZ35	P385R	rs765609303	TOLERATED	0.14	-0.37/decreased	-1.564	Neutral
10,69666562,1,C/G	B0QZ35	Q17E	rs772390347	TOLERATED	0.14	-1.38/decreased	-1.579	Neutral
10,69672655,1,G/C	B0QZ35	Q291H	rs759555466	TOLERATED	0.14	-1.17/decreased	-1.134	Neutral
10,69669105,1,G/C	B0QZ35	Q118H	rs776212608	TOLERATED	0.13	-1.92/decreased	-1.877	Neutral
10,69672447,1,C/T	B0QZ35	S222L	rs199649997	TOLERATED	0.13	-0.25/decreased	-1.509	Neutral
10,69667865,1,G/A	B0QZ35	G82R	rs758727461	TOLERATED	0.12	-0.26/decreased	-0.355	Neutral
10,69669131,1,A/G	B0QZ35	K127R	rs772640272	TOLERATED	0.12	-1.33/decreased	-2.53	Deleterious
10,69666607,1,A/G	B0QZ35	K32E	rs751410492	TOLERATED	0.12	0.31/increased	-1.657	Neutral
10,69669097,1,C/T	B0QZ35	P116S	rs775426483	TOLERATED	0.12	-1.84/decreased	-6.457	Deleterious
10,69672627,1,C/G	B0QZ35	S282C	rs748473217	TOLERATED	0.11	-0.44/decreased	-1.282	Neutral
10,69672524,1,G/A	B0QZ35	V248M	rs758444346	TOLERATED	0.11	-0.69/decreased	-0.538	Neutral
10,69669050,1,C/A	B0QZ35	P100Q	rs748394475	TOLERATED	0.1	-0.85/decreased	-1.547	Neutral
10,69672276,1,C/G	B0QZ35	P165R	rs777705431	TOLERATED	0.1	-0.76/decreased	-5.303	Deleterious
10,69669139,1,G/C	B0QZ35	V130L	rs745364339	TOLERATED	0.1	-0.48/decreased	-1.206	Neutral
10,69666595,1,G/A	B0QZ35	A28T	rs201340003	TOLERATED	0.09	-1.02/decreased	-1.057	Neutral
10,69676223,1,C/G	B0QZ35	P403R	rs749218988	TOLERATED	0.08	-0.77/decreased	-0.966	Neutral
10,69672519,1,C/G	B0QZ35	S246C	rs373331174	TOLERATED	0.08	-1.16/decreased	-0.839	Neutral
10,69676033,1,C/G	B0QZ35	L340V	rs751212678	TOLERATED	0.07	-0.67/decreased	-0.868	Neutral
10,69676204,1,A/C	B0QZ35	N397H	rs747251569	TOLERATED	0.07	-0.59/decreased	-1.742	Neutral
10,69669029,1,C/T	B0QZ35	P93L	rs757269120	TOLERATED	0.07	-1.34/decreased	-7.329	Deleterious
10,69672780,1,G/A	B0QZ35	R333Q	rs771135356	TOLERATED	0.07	-1.35/decreased	-2.059	Neutral
10,69669115,1,G/A	B0QZ35	A122T	rs200690371	TOLERATED	0.06	-0.03/decreased	-1.862	Neutral
10,69667848,1,A/C	B0QZ35	D76A	rs756786753	TOLERATED	0.06	-0.91/decreased	-3.303	Deleterious
10,69676215,1,A/C	B0QZ35	E400D	rs201327262	TOLERATED	0.06	-1.36/decreased	-0.816	Neutral
10,69676215,1,A/T	B0QZ35	E400D	rs201327262	TOLERATED	0.06	-1.36/decreased	-0.816	Neutral
10,69672530,1,G/T	B0QZ35	V250F	rs773456695	TOLERATED	0.06	-0.26/decreased	-0.799	Neutral
10,69667869,1,A/C	B0QZ35	D83A	rs17855430:C	DAMAGING	0.05	-1.35/decreased	-5.798	Deleterious
10,69672285,1,A/G	B0QZ35	H168R	rs779026786	DAMAGING	0.05	-1.31/decreased	-7.23	Deleterious
10,69672324,1,T/A	B0QZ35	V181D	rs1063111	DAMAGING	0.05	-1.59/decreased	-6.395	Deleterious
10,69666665,1,C/T	B0QZ35	A51V	rs141528984	DAMAGING	0.04	0.69/increased	-3.419	Deleterious
10,69676219,1,G/A	B0QZ35	E402K	rs778261267	DAMAGING *Warning! Low confidence.	0.04	-0.42/decreased	-1.024	Neutral
10,69672282,1,C/T	B0QZ35	P167L	rs201948258	DAMAGING	0.04	-0.72/decreased	-5.575	Deleterious
10,69666675,1,A/C	B0QZ35	Q54H	s116374368	DAMAGING	0.04	-1.4/decreased	-2.377	Neutral
10,69666675,1,A/T	B0QZ35	Q54H	rs116374368	DAMAGING	0.04	-1.4/decreased	-2.377	Neutral
10,69672374,1,T/C	B0QZ35	C198R	rs772656917	DAMAGING	0.03	-1.56/decreased	-8.169	Deleterious
10,69676279,1,G/A	B0QZ35	E422K	rs201112743	DAMAGING *Warning! Low confidence.	0.03	-0.95/decreased	-0.509	Neutral
10,69672290,1,C/T	B0QZ35	H170Y	rs751498023	DAMAGING	0.03	0.1/decreased	-2.752	Deleterious
10,69667835,1,A/G	B0QZ35	K72E	rs753398498	DAMAGING	0.03	-0.42/decreased	-2.77	Deleterious
10,69676156,1,A/C	B0QZ35	S381R	rs114572830	DAMAGING *Warning! Low confidence.	0.03	-0.49/decreased	-1.398	Neutral
10,69669116,1,C/G	B0QZ35	A122G	rs201635394	DAMAGING	0.02	-1.07/decreased	-2.995	Deleterious
10,69667871,1,A/C	B0QZ35	I84L	rs17855431	DAMAGING	0.02	-0.09/decreased	-1.848	Neutral
10,69669050,1,C/T	B0QZ35	P100L	rs748394475	DAMAGING	0.02	-0.33/decreased	-3.331	Deleterious
10,69676105,1,T/G	B0QZ35	S364A	rs772469360	DAMAGING	0.02	-1.07/decreased	-1.339	Neutral
10,69676123,1,A/G	B0QZ35	S370G	rs377449611	DAMAGING	0.02	-1.98/decreased	-2.004	Neutral
10,69676129,1,A/G	B0QZ35	S372G	rs759347614	DAMAGING	0.02	E	-2.414	Neutral
10,69672405,1,C/T	B0QZ35	T208I	rs750553699	DAMAGING	0.02	1.34/increased	-3.254	Deleterious
10,69669115,1,G/C	B0QZ35	A122P	rs200690371	DAMAGING	0.01	-0.12/decreased	-3.21	Deleterious
10,69666664,1,G/T	B0QZ35	A51S	rs777323664	DAMAGING	0.01	-0.35/decreased	-2.737	Deleterious
10,69667850,1,T/C	B0QZ35	C77R	rs779440453	DAMAGING	0.01	-1.55/decreased	-4.545	Deleterious
10,69676085,1,A/C	B0QZ35	D357A	rs778184510	DAMAGING	0.01	-2.58/decreased	-4.873	Deleterious
10,69676217,1,A/C	B0QZ35	D401A	rs151026272	DAMAGING *Warning! Low confidence.	0.01	-0.19/decreased	-1.941	Neutral
10,69667869,1,A/G	B0QZ35	D83G	rs17855430	DAMAGING	0.01	-2.13/decreased	-5.487	Deleterious
10,69669090,1,A/C	B0QZ35	E113D	rs771950281	DAMAGING	0.01	-0.78/decreased	-2.83	Deleterious
10,69669073,1,A/G	B0QZ35	I108V	rs149206117	DAMAGING	0.01	-0.86/decreased	-0.932	Neutral
10,69666592,1,A/G	B0QZ35	I27V	rs552023236	DAMAGING	0.01	-0.06/decreased	-0.823	Neutral
10,69672761,1,A/G	B0QZ35	K327E	rs199770148	DAMAGING	0.01	-0.33/decreased	-2.073	Neutral
10,69669148,1,C/T	B0QZ35	L133F	rs755327185	DAMAGING	0.01	0.05/increased	-3.628	Deleterious
10,69672308,1,C/T	B0QZ35	L176F	rs754951946	DAMAGING	0.01	0.36/increased	-3.79	Deleterious
10,69676160,1,T/C	B0QZ35	L382S	rs754362414	DAMAGING *Warning! Low confidence.	0.01	E	-2.203	Neutral
10,69676172,1,T/G	B0QZ35	M386R	rs750707792	DAMAGING *Warning! Low confidence.	0.01	-0.04/decreased	-1.467	Neutral
10,69676205,1,A/T	B0QZ35	N397I	rs116459300	DAMAGING *Warning! Low confidence.	0.01	0.29/increased	-2.884	Deleterious
10,69669097,1,C/A	B0QZ35	P116T	rs775426483	DAMAGING	0.01	-1.89/decreased	-6.707	Deleterious
10,69672239,1,C/G	B0QZ35	P153A	rs201348222	DAMAGING	0.01	-1.64/decreased	-7.352	Deleterious
10,69672239,1,C/A	B0QZ35	P153T	rs201348222	DAMAGING	0.01	-1.39/decreased	-7.319	Deleterious
10,69672270,1,G/A	B0QZ35	R163K	rs764179598	DAMAGING	0.01	-0.45/decreased	-2.743	Deleterious
10,69676088,1,C/T	B0QZ35	S358F	rs757624637	DAMAGING	0.01	0.21/increased	-3.193	Deleterious
10,69676105,1,T/C	B0QZ35	S364P	rs772469360	DAMAGING	0.01	-0.48/decreased	-1.779	Neutral
10,69676115,1,C/T	B0QZ35	S367F	rs768864413	DAMAGING	0.01	-0.32/decreased	-2.558	Deleterious
10,69676115,1,C/A	B0QZ35	S367Y	rs768864413	DAMAGING	0.01	-0.47/decreased	-2.064	Neutral
10,69676129,1,A/T	B0QZ35	S372C	rs759347614	DAMAGING	0.01	-0.82/decreased	-2.959	Deleterious
10,69666661,1,G/C	B0QZ35	V50L	rs200058231	DAMAGING	0.01	-0.01/decreased	-2.384	Neutral
10,69672320,1,G/A	B0QZ35	D180N	rs145326137	DAMAGING	0	-0.7/decreased	-4.738	Deleterious
10,69666668,1,G/A	B0QZ35	G52E	rs757804740	DAMAGING	0	-1.9/decreased	-6.479	Deleterious
10,69669152,1,T/C	B0QZ35	I134T	rs768051584	DAMAGING	0	-0.23/decreased	-4.51	Deleterious
10,69672238,1,A/G	B0QZ35	I152M	rs200021101	DAMAGING	0	-1.03/decreased	-2.226	Neutral
10,69672258,1,T/A	B0QZ35	I159K	rs140677498	DAMAGING	0	-0.37/decreased	-6.333	Deleterious
10,69672327,1,T/C	B0QZ35	I182T	rs1063112	DAMAGING	0	-0.4/decreased	-4.671	Deleterious
10,69669173,1,A/G	B0QZ35	K141R	rs756329197	DAMAGING	0	-1.54/decreased	-2.846	Deleterious
10,69669145,1,C/T	B0QZ35	L132F	rs199593180	DAMAGING	0	0.67/increased	-3.795	Deleterious
10,69669146,1,T/A	B0QZ35	L132H	rs766945174	DAMAGING	0	-0.73/decreased	-6.641	Deleterious
10,69669145,1,C/G	B0QZ35	L132V	rs199593180	DAMAGING	0	0.23/increased	-2.846	Deleterious
10,69672262,1,A/T	B0QZ35	L160F	rs761122062	DAMAGING	0	-1.43/decreased	-3.79	Deleterious
10,69666629,1,A/C	B0QZ35	N39T	rs767148239	DAMAGING	0	-0.32/decreased	-5.535	Deleterious
10,69669196,1,C/T	B0QZ35	P149S	rs267602551	DAMAGING	0	-0.6/decreased	-7.579	Deleterious
10,69672417,1,C/T	B0QZ35	P212L	rs201863201	DAMAGING	0	E	-3.214	Deleterious
10,69672779,1,C/T	B0QZ35	R333W	rs201647881	DAMAGING	0	-0.75/decreased	-4.292	Deleterious
10,69666625,1,C/T	B0QZ35	R38C	rs201583982	DAMAGING	0	-0.52/decreased	-7.38	Deleterious
10,69666626,1,G/A	B0QZ35	R38H	rs147909071	DAMAGING	0	-0.72/decreased	-4.613	Deleterious
10,69669164,1,C/T	B0QZ35	S138F	rs752958196	DAMAGING	0	-1.61/decreased	-5.693	Deleterious
10,69676106,1,C/T	B0QZ35	S364F	rs780449017	DAMAGING	0	-0.1/decreased	-2.214	Neutral
10,69666661,1,G/T	B0QZ35	V50F	rs200058231	DAMAGING	0	0.23/increased	-4.016	Deleterious

### Identification of damaging nsSNPs

The following bioinformatics tools have provided the supplied data to further detect the influence of 252 nsSNPs on the structure and function of the SIRT1 gene. Because the resulting values were lower than the Tolerance Index (0.05), the SIFT software revealed 94 nsSNPs to be intolerant ( Table 1).

Protein stability changed depending on which amino acid was substituted and 216 nsSNPs demonstrated a decline in stability based on DDG value received from I-Mutant server ( Table 1).

PROVEAN identified 77 nsSNPs as having a negative impact since the final score of the variations was lower than the specified value of threshold (-2.5).

**Structural and functional effect of nsSNPs**

**Figure 2.**
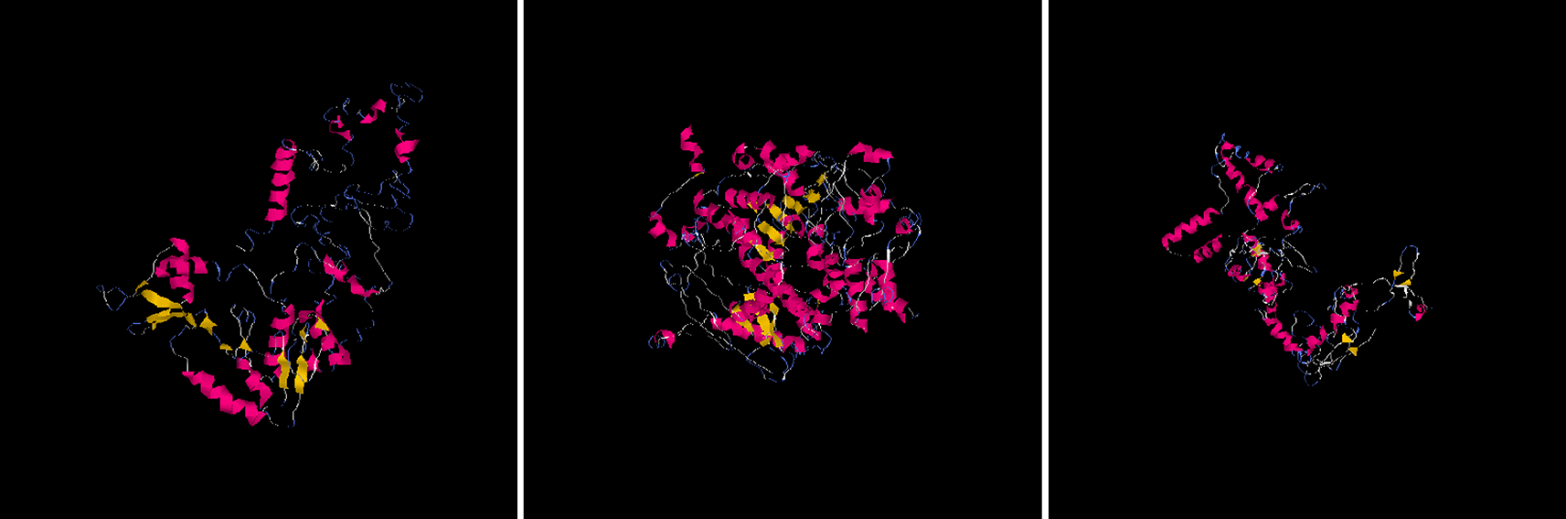
Predicted protein structure models by I Tasser of SIRT1 Gene for each SNPs rs778184510, rs76519031, rs199983221 respectively.

I-Mutant predicted the three (3) nsSNPs which played a role in decreasing SIRT1 stability (from each isoform - rs778184510, rs76519031, rs199983221), and they were selected for finding the impact of substitution of amino acid on structure and function of human protein (using Polyphen) and for the comparison of protein model (using I-Tasser). To generate the SIRT1 protein structure, SIRT1 protein sequences, single amino acid from the wild type, and mutations were uploaded to I-Tasser, which is the most accurate and sophisticated technique for predicting protein structure ( Figure 2). Then, using this technique, five models for each SIRT1 mutation and protein were produced.

When the native structure is known, TM score and RMSD can be used to compare the structural similarity of two structures. ^26^ The proposed TM score is supposed to solve the RMSD issue, which is prone to local errors. A local error (such as a mismatched tail) will raise the RMSD score even if the overall topology is good, since the RMSD measures the average distance between all residue pairs between two structures. The TM-score is insensitive to the local modelling error, nevertheless, because the short distance is weighted more severely than the long distance. A model with a proper topology is indicated by a TM-score and IT greater than 0.5, while a random similarity is indicated by a TM-score & IT less than 0.17 ( Table 2). These cut-offs are independent of the length of the protein.

**Table 2.** Results of Polyphen and I-Tasser analyses of three nsSNPs.

Protein ID	Residual change	Polyphen			I-Tasser		
		Score	Sensitivity	specificity	TM Score	RMSD values	C Score
rs778184510	D357A	0.983/Damaging	0.74	0.96	0.29±0.09	17.0±2.8Ǻ	-3.92
rs769519031	I223S	0.997/Damaging	0.27	0.98	0.42±0.14	14.6±3.7Ǻ	-2.55
rs199983221	I4T	1.00/Damaging	0.00	1.00	0.48±0.15	11.7±4.5Ǻ	1.93

By calculating a confidence score, or C-score, I-Tasser evaluates the accuracy of anticipated models. The convergence parameters from simulations of the structure assembly and the significance of threading template alignments are used to make this determination. A model with a high level of confidence also has a higher C-score. The C-score typically ranges from (-5,2).

### HOPE modelling for rs199983221

Isoleucine turned into threonine in position 4. The mutant residue was more compact than the wild-type residue. The mutant residue was also less hydrophobic than the wild-type residue. The mutation caused the hydrophobic contacts in the protein's core to disappear.

An overview of the protein is also displayed in the ribbon presentation ( Figure 3a). Additionally, there are five detailed pictures of the mutation site ( Figure 3b).

**Figure 3a.**
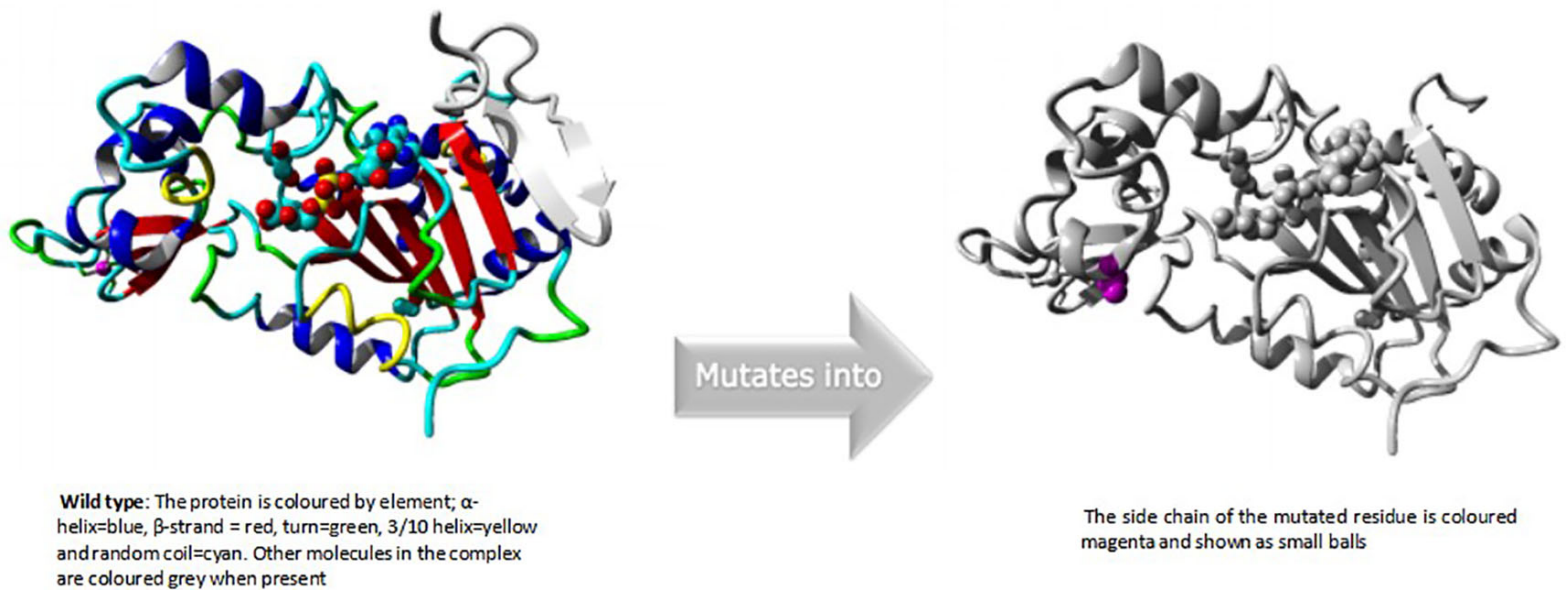
Overview of the protein in ribbon presentation (I4T).

**Figure 3b.**
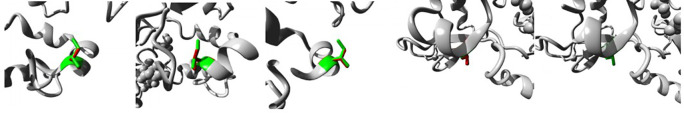
Close-up of the mutation. The protein is coloured grey, the side chains of both the wild-type and the mutant residue are shown and coloured green and red respectively. (I4T).

### HOPE modelling of rs769519031

In this case, isoleucine turns into serine at position 223. The mutant residue was more compact than the wild-type residue. The mutant residue was less hydrophobic than the wild-type residue. This could lead to a lack of interactions with the other genes The mutation may cause the proteins' surface-bound hydrophobic interactions with other molecules to disappear. ^19^

An overview of the protein is also displayed in the ribbon presentation ( Figure 4a). There are also five enlargements of the mutation location ( Figure 4b).

**Figure 4a.**
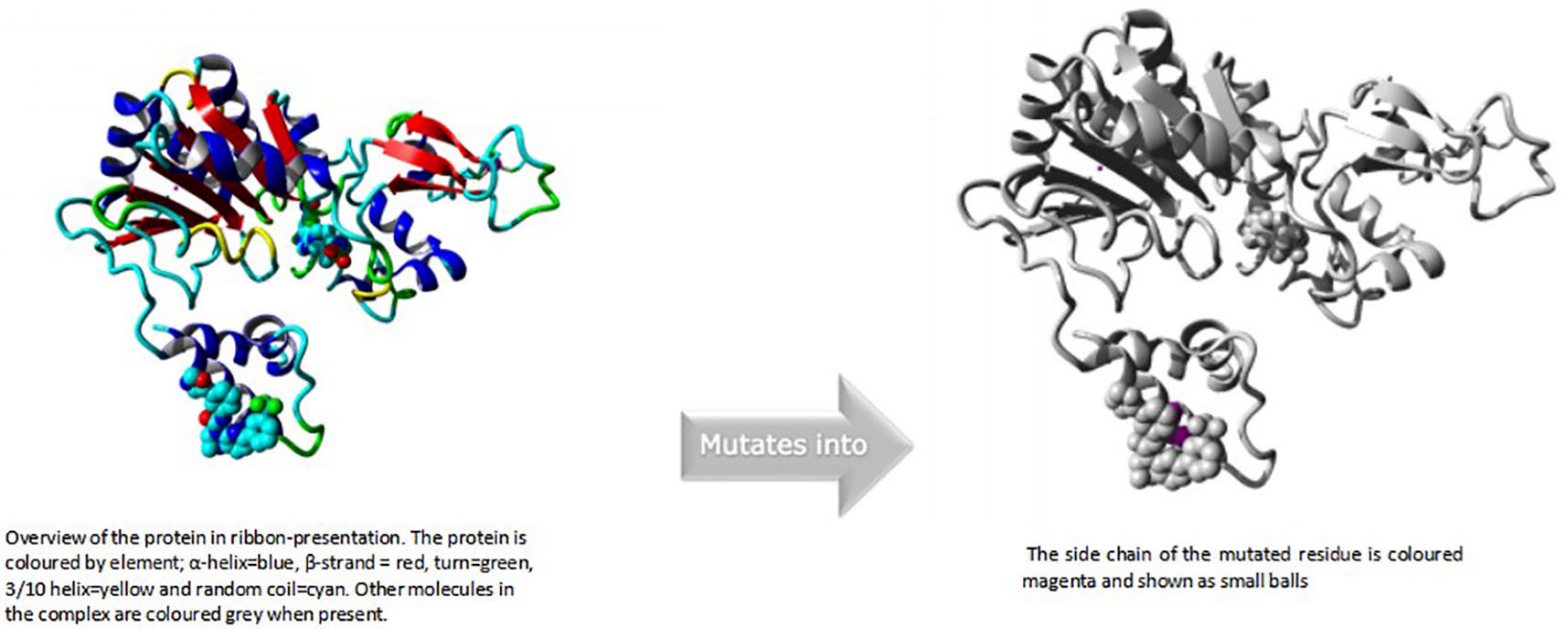
Overview of the protein in ribbon presentation (I223S).

**Figure 4b.**
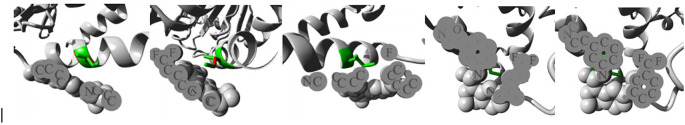
Close-up of the mutation. The protein is coloured grey, the side chains of both the wild-type and the mutant residue are shown and coloured green and red respectively (I223S).

### HOPE modelling of rs778184510

The mutant residue was smaller than the wild-type residue in this instance because alanine has replaced aspartic acid at position 357. In contrast to the wild-type residue charge, which was negative, the mutant residue charge was neutral. The mutant residue was more hydrophobic than the wild-type residue. The wild-type residue is expected to be located in its preferred secondary structure turn, according to the Reprof programme. The local conformation would only be slightly unstable since the mutant residue prefers a different secondary structure. The mutation places a more hydrophobic residue here. Hydrogen bonds may break as a result of this, and it may also prevent correct folding.

### Gene interactions

**Figure 5.**
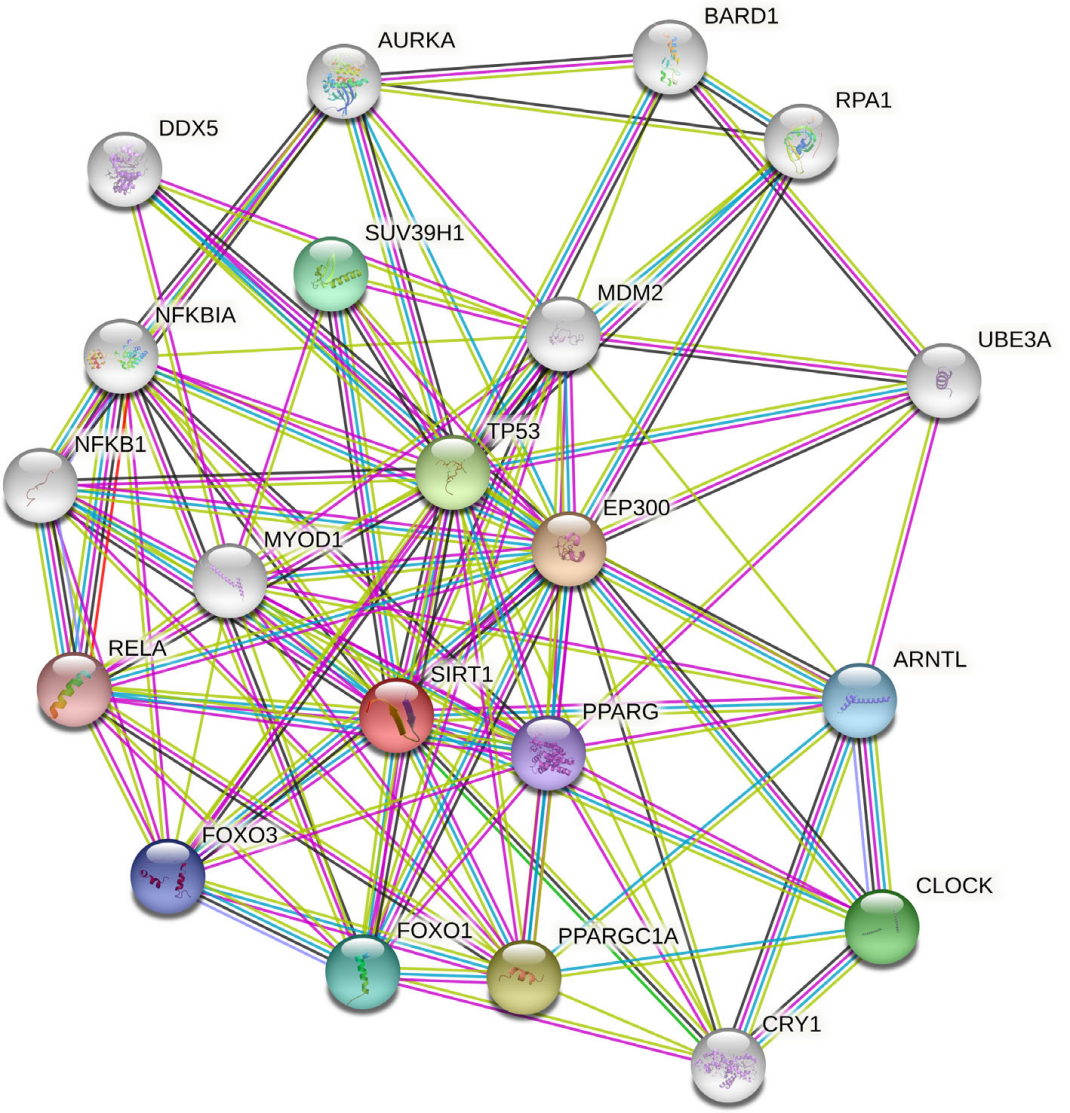
Gene interactions of SIRT1.

STRING revealed the physical interactions between SIRT1 and other genes in the gene's interactions. In its pathways, it interacted with NFKB1, NFKB1A, DDX5, AURKA, BARD1, RPA1, UBEBA, ARNTL,CLOCK,CRY1, PPARGC1A, FOXO1, FOXO3, RELA, MYOD1, SUV39H1, MDM2, EP300, PPARG and TP53 ( Figure 5). The query proteins and the initial line of SIRT1's interaction are represented by coloured nodes on the picture. White nodes are the second interactional shell. Protein-protein interactions are represented by edges. The edges of the known interactions are blue and pink. Others illustrate the predicted interplay between proteins. TP53 is more connected to and interdependent with SIRT1 than any other interaction on the list.

Numerous studies have been done in the past to determine the connection between the SIRT1 gene's polymorphism and a number of conditions, such as cancer, inflammation, obesity, diabetes, and cardiovascular and neurological illnesses. The most harmful nsSNPs in the SIRT1 gene that may be crucial in the development of certain disorders have been explored in this work.

The SIRT1 gene has 252 nsSNPs, according to our findings. The present study's SIFT findings revealed that the SIRT1 protein contains 94 harmful nsSNPs, 66 of which are detrimental as indicated by PROVEAN.

Provean scores were -4.873, -4.47, -4.39 for rs778184510, rs769519031 and rs199983221 respectively which were higher compared to other SNP’s and were chosen from each isoform of SIRT1 protein. These three nsSNPs, which cause high risk of altering normal functioning of SIRT1 gene, were selected for further evaluation based on the I-Mutant value, from each isoform of the SIRT1 protein.

D357A, I223S, and I4T’s respective Polyphen2 scores, which range from 0 to 1, were 0.983, 0.997, and 1.00, respectively; all three were classified as having probable damage by Polyphen2. Protein structure and functional activity depend on protein stability. ^27^ Thus, I-Mutant, which was used to assess the stability of protein, demonstrated the protein stability for D357A, I223S, and I4T as -2.58, -2.25 and -2.2, respectively, as the lowest values. Thus, these three SNPs affect the function and structure of SIRT1 protein.

By determining the RMSD values and TM scores for each mutant model, we expanded our analysis. While RMSD aids in calculating the average distance between the carbon backbones of wild and mutant models, the TM score is utilised to assess the topological similarity between wild- and mutant-type models. ^20^^,^^21^ The mutant model D357A demonstrated a greater RMSD value, which had a greater deviation from the wild type compared to the other two mutant models. To further establish the detrimental impacts of these nsSNPs, the SIRT1 protein structure was determined using I-Tasser, and the protein's FASTA sequence served as the sole input. Using I-Tasser, the prototypes are acquired, and the protein simulation is carried out. Following the introduction of the mutant models to the HOPE server, the server generated the effects of mutations on the contacts and the structural placement.

Mutations can affect a protein's stability, structure, and ultimately, function. Mutations are components of the "raw material" of evolution. The majority of, if not all, protein mutations are eliminated by negative, purifying selection, which lowers the probability of subsequent adaptations.

Because of this, under the influence of positive selection, only a small portion of all potential mutations will be resolved to take on a new function. Randomness or "neutral drift" might theoretically cause neutral mutations to randomly correct in small populations. At the level of the organism, the consequences of mutations on fitness are complicated and seldom ever correlate to the characteristics of a single gene or protein. Several levels of redundancy, resilience, and backup decrease the impact of numerous mutations. Understanding and predicting the impact of mutations at the organismal level present important problems for evolutionary biology. ^28^^,^^29^

The stability of the proteins is influenced by the quantity of functional protein present. According to previous research, stability and folding effects are responsible for 80% of the negative consequences of pathogenic mutations. ^30^ Protein dysfunctionalization is mostly caused by mutations that reduce the amount of soluble, functional proteins over a specified threshold (or DDG value). ^30^ Experimental studies on a variety of proteins indicated that between 33 and 40% of the time, a detrimental mutation is likely to occur. ^29^ As mutation rates increase, protein fitness therefore substantially decreases. When five mutations are introduced into a protein, its fitness is decreased by 20%.

Protein evolution rates, and maybe even the rates at which entire organisms evolve, seem to be primarily (though surely not solely) influenced by stability, ^31^^,^^32^ particularly but not entirely in connection with the acquisition of new functionalities. Stability appears to be the primary (though surely not the only) driver of how rapidly proteins change, despite the fact that a protein's starting stability might mitigate some of the destabilising effects of mutations.

For a small number of proteins, experimental datasets are frequently made accessible, and they generally focus on changes in mutation thermodynamic stability (DDG values). Recent developments in computing have made it possible for researchers to predict the DDG values of certain protein mutations. Some prediction methods strongly rely on sequence, whereas others mostly rely on three-dimensional structures. ^33^^,^^34^

New protein functions cannot be developed because of the destabilising impact of mutations. Neutral or non-adaptive mutational drifts have been found to be less disruptive and to occur frequently at buried residues as compared to new function or adaptive mutations. ^35^

The mutant study shows the decreased thermodynamic stability of the proteins, regardless of whether SIFT and Provean examinations of SNPs in the leptin and leptin receptor genes suggest that they are detrimental or tolerated. This might have an impact on how leptin and leptin receptor proteins function. This conclusion supports previous studies linking leptin, leptin gene polymorphisms, and the incidence of depression in obese individuals.

Despite several studies relating SNPs in different genes to a number of disorders, computational analysis of the functional effects of SNPs in SIRT1 is still lacking. To determine whether an amino acid change will have an impact on protein function, the SIFT technique examines sequence homology across related genes and domains across evolution. The physical-chemical properties of the residues of amino acids are also considered. According to estimates, SIFT has error rates of 31% and 20% for false negatives and positives, respectively. When amino acid changes are used as the test set, SIFT is roughly 80% effective in benchmarking trials and is thought to significantly reduce the residual activity of the variant protein.

However, utilizing SIFT and Provean, it is now feasible to analyse gene polymorphisms and forecast how a mutation will alter a protein's functionality. Since most disease mutations have an effect on protein stability, I-Mutant assessed the stability of the mutant proteins.

To find, characterize, validate, and predict the functional consequences of harmful non-synonymous SNPs (nsSNPs) in the interleukin-8 gene, Dakal *et al.* carried out a comparable. ^36^

It may also be deduced that all three of the SIRT1 gene's most harmful nsSNPs eventually interfere with and disrupt the normal function of other expressive genes. Based on their interaction patterns and their correlation profiles with numerous diseases and their pathways, SIRT1 is involved in pathways with genes such as NFKB1, NFKB1A, DDX5, AURKA, BARD1, RPA1, UBEBA, ARNTL, CLOCK, CRY1, PPARGC1A, FOXO1, FOXO3, RELA, MYOD1, SUV39H1, MDM2, EP300, PPARG and TP53, which, therefore indicate its importance. ^37^

## Conclusions

The SIRT1 protein is essential for the development of numerous diseases, including cancer, inflammation, diabetes, obesity, and cardiovascular and neurological conditions. Therefore, to elucidate this domain’s function, studying its structural conformation is crucial. This *in silico* examination of the functional SNPs in SIRT1 sheds light on the possible damage that the nsSNPs could do to the protein. In this work, it was predicted for the first time how nsSNPs will affect the structure and function of the SIRT1 protein. The three nsSNPs (D357A, I223S, and I4T) in the SIRT1 gene were the most detrimental alterations, according to our research. Our findings will be a crucial reference point for research into prospective diagnostic and therapeutic approaches, because this protein has been linked to several disorders, necessitating large-scale clinical trials and investigations based on experimental mutational validation.

## Author contributions

Desy TM and Usha Adiga designed the research, performed softwares and wrote the manuscript; Tirthal Rai and Sachidananda Adiga, Vijith Shetty revised the manuscript and involved in data analysis. All authors read and approved the fnal manuscript.

## Data Availability

Biostudies: Identification Of The SIRT1 Gene's Most Harmful Non-Synonymous SNPs And Their Effects On Functional And Structural Features- An Insilico Analysis; Accession number: S-BSST944. https://identifiers.org/biostudies: S-BSST944 This project contains the following underlying data
-
Table 1: SIFT, I mutant analysis, Provean for the nsSNPs of SIRT1 Gene -
Figure 1: Sorting of nsSNPs of SIRT1 Data are available under the terms of the Creative Commons Attribution 4.0 International license (CC-BY 4.0).
