# Supplementary material for: Identification of the SIRT1 gene's most harmful non-synonymous SNPs and their effects on functional and structural features-an in silico analysis
Source: F1000Res. 2024 Jan 19;12:66. Originally published 2023 Jan 16. [Version 2] doi: 10.12688/f1000research.128706.2 (PMC10822041; doi:10.12688/f1000research.128706.2)
Supplement: Peer review history

# Reviewer report: Reviewer response for version 2

**DOI:** 10.5256/f1000research.161353.r239165

**Published:** 2024-01-27

**Authors:** Bhushan L. Thakur

**Affiliations:** 1 DNA replication group, DTB, NCI/NIH, National Cancer Institute, Bethesda, Maryland, USA

The authors have addressed all my concerns.

Is the work clearly and accurately presented and does it cite the current literature?

No

If applicable, is the statistical analysis and its interpretation appropriate?

Yes

Are all the source data underlying the results available to ensure full reproducibility?

Partly

Is the study design appropriate and is the work technically sound?

Yes

Are the conclusions drawn adequately supported by the results?

Yes

Are sufficient details of methods and analysis provided to allow replication by others?

Partly

Reviewer Expertise:

SIRT1 biology, DNA replication, Genomics.

I confirm that I have read this submission and believe that I have an appropriate level of expertise to confirm that it is of an acceptable scientific standard.

# Reviewer report: Reviewer response for version 1

**DOI:** 10.5256/f1000research.141323.r224496

**Published:** 2023-12-19

**Authors:** Bhushan L. Thakur

**Affiliations:** 1 DNA replication group, DTB, NCI/NIH, National Cancer Institute, Bethesda, Maryland, USA

The study provides a comprehensive analysis of single nucleotide polymorphisms (SNPs) in the SIRT1 protein, identifying the three most deleterious non-synonymous SNPs from the NCBI database. The objective of this research is to identify potentially harmful nsSNPs for SIRT1, which may potentially impact disease biology. The study is executed and written well.

 Below are suggested changes to improve clarity and structure:

1. The introduction should include citations to the most recent literature.
2. The methods section provides a concise outline of the collection and analysis of non-synonymous SNPs from the dbSNP site using bioinformatic tools. However, to enhance transparency and reproducibility, the authors should include additional details about the specific criteria or thresholds used to select deleterious nsSNPs.
3. In Figure 1, the method flowchart seems to indicate that the authors used SIFT, Provean, and I-Mutant sequentially to identify the final 66 nsSNPs. However, based on the method description and Table 1, it appears that they utilized commonality between high-score predictions from the three tools to identify the top three hits. The authors should clarify this in the method to reduce ambiguity and make necessary changes in Figure 1.
4. Based on Table 1 and the author's selection criteria for SNP selection, it is unclear why the authors did not shortlist the following three SNPs: D83G (rs17855430), P116T (rs775426483), and G52E (rs757804740). The authors should consider including the variants, G52D (rs757804740) and G52E (rs757804740), in the current manuscript or explain their exclusion.
5. The conclusion succinctly summarizes the most damaging mutations, if possible authors are encouraged to explicitly state the potential implications of these findings for understanding the pathogenesis of various disorders, any reports that cite identification of these SNP’s, and connecting them back to the broader significance mentioned in the introduction.

Minor comments

 - Correct the typo on page 5: " Isofrom a (NP_036370.2) comprised 597 nsSNPs, " should be isoform.

 - Verify the discrepancy on page 16: "PROVEAN identified 77 nsSNPs as having…" (Figure 1 shows 76).

 - Correct the typo on page 16: "..rs778184510, rs76519031, rs199983221)…" should be rs769519031.

 - Add a key for line colors connecting proteins in Figure 5.

 - Verify and provide a reference or criteria for the claim about I-Tasser in the statement: "...mutations were uploaded to I-Tasser, which is the most accurate and sophisticated technique for predicting protein structure (Figure 2)." Consider modifying it to "...one of the most..." if needed.

 -Please clarify, what is “E” in Table 1 in DDG column.

Is the work clearly and accurately presented and does it cite the current literature?

No

If applicable, is the statistical analysis and its interpretation appropriate?

Yes

Are all the source data underlying the results available to ensure full reproducibility?

Partly

Is the study design appropriate and is the work technically sound?

Yes

Are the conclusions drawn adequately supported by the results?

Yes

Are sufficient details of methods and analysis provided to allow replication by others?

Partly

Reviewer Expertise:

SIRT1 biology, DNA replication, Genomics.

I confirm that I have read this submission and believe that I have an appropriate level of expertise to confirm that it is of an acceptable scientific standard, however I have significant reservations, as outlined above.

# Response

**Published:** 2024-01-11

**Authors:** Usha Adiga

**Affiliations:** KS HEGDE MEDICAL ACADEMY, India

**Comment 1:** **The introduction should include citations to the most recent literature.**

 Answer: The latest references will be included respectively.

**Comment 2:** **The methods section provides a concise outline of the collection and analysis of non-synonymous SNPs from the dbSNP site using bioinformatic tools. However, to enhance transparency and reproducibility, the authors should include additional details about the specific criteria or thresholds used to select deleterious nsSNPs.**

 Answer: All three tools provide valuable insights into the functional consequences of genetic variations, their methodologies and focuses differ. SIFT predicts the functional impact of amino acid substitutions based on sequence conservation. It evaluates how well a particular amino acid is conserved across different species. PROVEAN predicts the impact of protein sequence variations using a combination of sequence homology and structural information. It considers the alignment of the query sequence with homologous sequences and the predicted structure changes caused by the variation. I-Mutant focuses on predicting the impact of amino acid substitutions on protein stability. It employs an energy-based approach, evaluating changes in free energy upon mutation to predict whether a mutation destabilizes or stabilizes the protein structure.

**Comment 3:** **In Figure 1, the method flowchart seems to indicate that the authors used SIFT, Provean, and I-Mutant sequentially to identify the final 66 nsSNPs. However, based on the method description and Table 1, it appears that they utilized commonality between high-score predictions from the three tools to identify the top three hits. The authors should clarify this in the method to reduce ambiguity and make necessary changes in Figure 1.**

 Answer: In the SIRT1 gene, three isoforms were identified, resulting in a total of 252 identified Single Nucleotide Polymorphisms (SNPs). Among them, SIFT software identified 94 non-synonymous SNPs (nsSNPs) as intolerant (using a cutoff value of <0.05). Subsequently, these 94 intolerant nsSNPs underwent Provean analysis, revealing 67 nsSNPs as harmful (with a cutoff value of <-2.5). The selected 67 nsSNPs then underwent I-Mutant analysis, which indicated 58 nsSNPs with decreased stability (using a cutoff value of 0). From this subset, we selected three nsSNPs from each isoform, respectively, based on decreasing protein stability.

 The figure will be modified accordingly.

**Comment 4:** **Based on Table 1 and the author's selection criteria for SNP selection, it is unclear why the authors did not shortlist the following three SNPs: D83G (rs17855430), P116T (rs775426483), and G52E (rs757804740). The authors should consider including the variants, G52D (rs757804740) and G52E (rs757804740), in the current manuscript or explain their exclusion.**

**Answer: **We have chosen one non-synonymous Single Nucleotide Polymorphisms (nsSNPs) from each of the three isoforms, specifically focusing on those identified as more damaging and exhibiting decreased stability.

**Comment 5:** **The conclusion succinctly summarizes the most damaging mutations, if possible authors are encouraged to explicitly state the potential implications of these findings for understanding the pathogenesis of various disorders, any reports that cite identification of these SNP’s, and connecting them back to the broader significance mentioned in the introduction.**

 Answer: There is no existing literature on the specified SNPs of SIRT1 gene.

**Minor comments**

**- Correct the typo on page 5: " Isofrom a (NP_036370.2) comprised 597 nsSNPs, " should be isoform.**

 Answer: Corrected

**- Verify the discrepancy on page 16: "PROVEAN identified 77 nsSNPs as having…" (Figure 1 shows 76).**

**Answer: **corrected the number of SNPs after rechecking to 67

**- Correct the typo on page 16: "..rs778184510, rs76519031, rs199983221)…" should be rs769519031.**

**Answer: **Corrected

**- Add a key for line colors connecting proteins in Figure 5.**

**Answer: **The explanation will be included in the text.

****In evidence mode, an edge may be drawn with up to 7 differently colored lines - these lines represent the existence of the seven types of evidence used in predicting the associations. Red lines indicate the presence of fusion evidence, green lines represent neighborhood evidence, blue lines suggest cooccurrence evidence, purple lines correspond to experimental evidence, yellow lines denote text mining evidence, light blue lines signify database evidence, and black lines represent co-expression evidence.

**- Verify and provide a reference or criteria for the claim about I-Tasser in the statement: "...mutations were uploaded to I-Tasser, which is the most accurate and sophisticated technique for predicting protein structure (Figure 2)." Consider modifying it to "...one of the most..." if needed.**

 Answer: The statement is corrected accordingly.

**-Please clarify, what is “E” in Table 1 in DDG column.**

**Answer: **E represents error from the analysis report.

 Thank you for the valuable comments. All the corrections and suggestions will be incorporated in the manuscript accordingly.

# Reviewer report: Reviewer response for version 1

**DOI:** 10.5256/f1000research.141323.r162701

**Published:** 2023-02-21

**Authors:** Laxminarayana Kurady Bairy

**Affiliations:** 1 Department of Pharmacology, RAK College of Medical Sciences, RAK Medical and Health Sciences University, Ras al Khaimah, United Arab Emirates

- The article is well presented. The aims and objectives are clearly mentioned.
- The introduction part is well written. However, the cited references are before 2016. In fact, the majority are before 2010. There is no update on the recent developments in the field. Hence, the introduction needs to be rewritten with recent references.
- The study design is appropriate and the work is technically sound. The methodology used is clear, described in detail and is enough for anyone to repeat the experiment. The statistical method is appropriate.
- The source data is not available in the manuscript provided. However, it is not customary to give sorce data in the manuscript. Most of the journals do not ask for source data. However, if it is required, editorial can ask for the same.
- There is some ambiguity in the conclusion drawn. It is a bit vague. This needs to rewritten to make it more lucid.

Is the work clearly and accurately presented and does it cite the current literature?

No

If applicable, is the statistical analysis and its interpretation appropriate?

Yes

Are all the source data underlying the results available to ensure full reproducibility?

No source data required

Is the study design appropriate and is the work technically sound?

Yes

Are the conclusions drawn adequately supported by the results?

Partly

Are sufficient details of methods and analysis provided to allow replication by others?

Yes

Reviewer Expertise:

My area of research includes pharmacogenomics, wound healing and neurogenerative disorders.

I confirm that I have read this submission and believe that I have an appropriate level of expertise to confirm that it is of an acceptable scientific standard.

# Response

**Published:** 2023-03-08

**Authors:** Usha Adiga

**Affiliations:** KS HEGDE MEDICAL ACADEMY, India

Introduction will be modified with recent references.

 Conclusion will be modified.
